# Use of Decompressive Craniectomy in Tbi Patients in a London Major Trauma Centre

**DOI:** 10.1186/2197-425X-3-S1-A489

**Published:** 2015-10-01

**Authors:** C Lopez Soto, G Sivasubramaniam, J Dawson, R Maharaj

**Affiliations:** Norfolk & Norwich University Hospitals, Anaesthetics & Intensive Care, Norwich, United Kingdom; King's College Hospital, Critical Care, London, United Kingdom

## Introduction

Traumatic Brain Injury(TBI) is a serious health problem resulting in 1x10^6^ Emergency admissions per annum in the UK [[1]].39% of patients with severe TBI, or GCS less than 8, die and 60% survive with unfavourable outcomes [[2]].The aim of TBI management is to minimise secondary brain injury from cerebral oedema to maintain brain oxygen delivery, which may be achieved via medical or surgical management, latest by using decompressive craniectomy (DC) in an attempt to reduce intracranial pressure (ICP).However, no current guidelines on its use have been published and its use remains controversial [[3]].

## Objectives

Primarily, to describe the incidence and indication for DC in a Major Trauma Tertiary Centre.Secondly, to further evaluate ICU and in-hospital mortality rates in those patients treated medically vs those who were decompressed.

## Methods

We retrospectively collected the following data in all TBI patients admitted to our institution over a 3yr period from July 2011-July 2014:gender, age, APACHE score, presenting GCS. For those patients who received DC, we recorded the indication to proceed to surgery. ICU and Hospital mortality was recorded in all patients. Glasgow Outcome Score was recorded in all that was documented.

Descriptive statistics were used to report data.

## Results

Over the3 year period,481 patients were admitted to the ICU with a diagnosis of TBI.Demographic data is shown on Table 1. Forty-seven patients (9.77%) received DC. in 25(53.19%),decision was based on initial scan and examination and in 22(46.81%) on ICP refractory to medical managementICU mortality was 25.53% of those who got DC compared to 12.89% of all patients medically treated and both ICU and Hospital mortality were higher in patients who received DC.Unfortunately, only 47 patients(9.8%) had a documented GOS.

**Table 1:** *Patient demographics.*

Gender	Male 370 (76.92%) Female 111 (23.07%)
Age (yrs)	46.8
APACHE Score	15.28
First GCS documented	15 - 13: 107 (22.25%) 12 - 9: 101 (20.99%) ≤ 8: 267 (55.51%) Not recorded: 6 (1.25%)

## Conclusions

Use of DC in our institution is in keeping with current published data [[4, 5]]. Both ICU and hospital mortality were higher in patients who received DC than those medically treated. Although this may be attributed the former being more severely injured, we think that further studies need to be done.Unfortunately, the lack of documented data on GOS rendered us unable to analyse this feature of management.As a TBI follow-up clinic has been set up in our trust, we hope to study the effect of DC on neurological outcome in the near future.
